# Supplementary material for: Foldy: An open-source web application for interactive protein structure analysis
Source: PLoS Comput Biol. 2024 Feb 2;20(2):e1011171. doi: 10.1371/journal.pcbi.1011171 (PMC10866462; doi:10.1371/journal.pcbi.1011171)
Supplement: Attachment — Submitted filename: 231222 Response to Reviewers.pdf [file pcbi.1011171.s002.pdf]

Dear Reviewers,

Thank you for your valuable feedback on our manuscript, "Foldy: an open-source web application for interactive protein structure analysis". We have addressed your remaining comments and we hope you agree that the manuscript is about ready for submission. Specifically:

- **We have added more detailed instructions** (and back up instructions) for reserving a GPU-accelerated VM in Google Cloud, which can be found in our new release: https://github.com/JBEI/foldy/releases/tag/v1.0.1. Note that, unfortunately, there is no automatic approach for finding the availability of GPU-accelerated VMs. GPU availability these days is low, and Google does not offer an easy way to see the availability of different GPUs in different regions. Therefore the best approach is to try a few different GPUs in a few different zones, and at different times, which we have described in more detail.
- **We corrected the errors in Fig 3 and Fig 4c.**
- **We clarified the "???" in Table 1.** You are correct, it was meant to imply that the tool was unavailable to analyze.

A line by line response to the reviewer's comments is below. We believe that these revisions address the concerns raised by the reviewers, and would be grateful if you would consider re-evaluating the manuscript.

Thank you again for your time.

Regards,
The authors of "Foldy: an open-source web application for interactive protein structure analysis"

Reviewer Comments and Author Responses
Reviewer #1: Thank you for taking your time to add the easier option Foldy-in-a-box deployment. It's also much clearer that the development option is for interface only.

Thank you!

Reviewer #2: Foldy is a useful interface that bridges the gap between experimental structural biologists and advanced AI-based protein structure prediction and docking models. Its user-friendly design and minimal computational resource requirements make it especially beneficial for structural biologists who may have limited coding experience or computational resources.

The revised version is much better prepared for publication. Each example is better introduced, the hypothesis is clear, and the method is easy to understand and reproduce. The inclusion of the comparison table shows the advantage of Foldy clearly. The updated instructions on GitHub are better described and easier to follow. However, there are minor issues that need to be addressed before it can be accepted for publication.

Issue:
When I tried to deploy Foldy-in-a-box on Google Cloud, the specified configuration, a n1-highmem-8 VM instance with 1 nvidia-tesla-t4 accelerator(s) was unavailable. It would be nice if there were alternative configurations for users to choose from.

We added more detailed instructions for deploying to a Google Cloud VM when there are few resources available. More details above.

Minor Revisions:
Figure 3: The caption mentions "L281W" while the figure shows "L321W."

Thank you for catching this error! We have corrected it.

Figure 4c: The left "PP_0765" should be "PP_0766," and the bottom "PP_0766" should be "PP_2019."
Table 1: The "???" on the row of BioNeMo is unclear. Does it mean that it is unavailable to analyze?

Thank you for catching this one as well!
