# Supplementary material for: Foldy: An open-source web application for interactive protein structure analysis
Source: PLoS Comput Biol. 2024 Feb 2;20(2):e1011171. doi: 10.1371/journal.pcbi.1011171 (PMC10866462; doi:10.1371/journal.pcbi.1011171)
Supplement: Attachment — Submitted filename: 230914 Response to Reviewers.docx [file pcbi.1011171.s001.docx]

Dear Reviewers,

Thank you for your time in reviewing the manuscript for our work on Foldy. Your comments were constructive and we feel that the manuscript and the tool itself have improved in response. We are especially appreciative of Reviewer’s efforts to set up the deployment, which is not a small task, and revealed a number of specific code bugs.

We believe we have addressed the reviewer’s comments. We address each comment (red-lined) below, and summarize the major changes since your last review according to three themes from your feedback:

1. Foldy was too hard to deploy.
   a. The old deployment was based on Kubernetes, which is not commonly used in academic labs. In addition to being a generally hard to use platform, Kubernetes also proved less portable than the authors initially realized, leaving the final deployment process laborious. In response to reviewer feedback, we made significant changes to allow Foldy to be more flexible in its deployment. Building on this flexibility, we added another well defined deployment option: Foldy-in-a-box. Foldy-in-a-box is a Foldy deployment on a standard Google Cloud Compute VM. Users in our lab were able to set this Foldy type up in less than 10 minutes. We believe this deployment option will make Foldy accessible to individuals and labs which don’t have dedicated IT teams, and we believe it has improved the applicability of Foldy.
2. There was insufficient comparison to other tools in this space.
   a. We have now enumerated a number of comparable open-source and closed-source platforms which run AlphaFold. It is surely an inexhaustive list but we believe it incorporates the major players which were mentioned by the reviewers. We compare the tools on a number of dimensions including maximum structure size, storage of structures, secondary tools, etc.
3. The case studies were presented unscientifically and the purpose of their inclusion was not clear.
   a. We have done more to frame the case studies and added relevant gene & organism details to tell a more complete story. We have also made some of the structures available through Foldy.

Please note also that most of the figures have been replaced, as we received feedback from the editor that screenshots are a potential violation of CC BY 4.0 policy.

Thank you for your time and we look forward to your responses.

Regards,

The authors of "Foldy: an open-source web application for interactive protein structure analysis"

**Reviewer Comments and Author Responses**

Reviewer #1: This is potentially a very useful application to allow users to predict AlphaFold structures in their local servers or via Google clouds. I created issues in Foldy GitHub pages when I had problems. However, after three weeks, I still can not use it to predict AlphaFold structures. I am sure most users will have the same experience if they try it.

The authors should spend significant effort to make sure users can use the methods to run their own structure prediction. I have the following comments.

1. If the local deployment is used for testing purpose and can not predict structures, authors should either describe how to use it for testing, or don't mention it all. My first impression is that I can use it to predict structures, but can't use the analysis tools.

We renamed the nonfunction deployment option from “Local” to “Development” to make its lack of functionality more clear, and added more documentation about this deployment option. We also included a summarial table in the README which states that the Development deployment option has no tools.

2. The steps to set up production deployment could be mentioned in the main paper. They are more important than the use cases. The supplementary should describe in more details about the steps so that other people can reproduce it. It should include some alternatives if something goes wrong.

We have addressed this feedback in a few ways:

- The Foldy-in-a-box deployment option dramatically simplifies deployment for labs which do not wish to do a Helm / Kubernetes deployment. Additionally, it includes more documentation for debugging when the deployment fails.
- Deployment options are discussed in the main text.

As a general comment, we think Foldy-in-a-box is the appropriate deployment option for most labs. These authors have realized that it was impractical to share instructions for setting up and configuring a Kubernetes cluster for the public. We have left the Helm deployment option written up, as a guide for Kubernetes administrators who wish to set up a more scalable instance of Foldy. But maintenance of a comprehensive Kubernetes deployment instruction manual with debugging instructions is, we have come to believe, beyond the scope of this article.

3. I spend three weeks and only get to step 6. Here are some comments about the application:

We are extremely grateful for this level of code review and for the bug reports.

1) Typo "]" in the file foldy/values.yaml: postgresql://postgres:${GOOGLE_SQL_DB_PASSWORD}@]${GOOGLE_SQL_DB_PRIVATE_IP}/postgres
Fixed.

2) Add the step to install yq. I used “pip3 install yq” and it actually is “jq”. I have to replace “yq eval” with “yq -r”.

Added to the instructions. Frankly we don't fully understand why installation required jq instead of yq. Hopefully the yq installation instructions will work for you and other users in the future.

3) I used “us-east1” from the beginning. So “us-central1”/”us-central1-c” should be a variable.

Done.

4. The value of the paper is to allow users to use your method to set up their own servers. I am sure they will have many questions. A timely response is crucial.

Understood.

5. If possible, you could include ESMFold in local deployment.

We did not reach this goal. We hope the Foldy-in-a-box deployment may lessen the need for a local deployment.

Reviewer #2: The presented paper offers an accessible web interface for protein structure prediction and ligand docking, employing advanced AI-based tools in structural biology, including AlphaFold and DiffDock. The tool can either be hosted on the provided cloud server or local devices, expanding its accessibility to biologists, even those with limited software expertise. The showcased case studies underscore the wide utility of this interface for ligand binding prediction, predicted structure evaluation, and protein-protein interaction prediction. This paper introduces a powerful tool for protein structure analysis.

Considering the potential impact of this article, the manuscript necessitates significant revisions as follows:

Major Revisions:

1. Results – Unsupported Advantage Claim: The assertion in the abstract that "Foldy enables scientists to predict the structure of proteins and complexes up to 3000 amino acids" lacks supporting evidence in the results, calling into question the stated benefit of the tool.

We have compared a number of comparable tools on important axes, and experimented on all for the ability to predict structures of different sizes.

2. Results – Absence of Benchmark Comparison: The paper acknowledges ColabFold and AlphaFold-Colab as benchmark applications for protein structure prediction, and AutoDock Vina and DiffDock for ligand docking, yet fails to provide a rigorous quantitative comparison to these benchmarks. It is crucial for the Scalability section to convincingly demonstrate how Foldy overcomes these benchmarks' limitations.

We measured and compared the maximum complex size of different AlphaFold running tools. We believe the real advantage of Foldy over ColabFold and AlphaFold-Colab is in the qualitative ability to store structures and to access multiple tools in a single interface. We have tried to clarify this distinguishing factor in the main text.

3. Discussion – Informal Case Studies: The case studies lack clear objectives, resembling experimental notes rather than comprehensive research investigations. Key details like target protein names, sequence lengths, processing methods, and benchmark comparison results should be explicitly presented. Furthermore, it appears that the case studies could potentially be replicated using benchmark tools, undermining the claim of Foldy's unique capabilities.

We have added some more details to the presented case studies, and added more context for their inclusion as inspirational use cases. We have also made some of the structures public, to allow readers to learn more about the structures and sequences.

We agree that these analyses could all be done with other tools - Foldy does not introduce any new bioinformatics tools. Rather, in all three examples, the biologists *would not have used* the standalone tools because they are too inaccessible. Our users, for example, would not use DiffDock or Autodock Vina because they require use of the command line, which is an imposition for many primarily wet lab biologists.

Minor Revisions:

1. Results – Figure 1: Detailed information about the dashboard in relation to the third case study would enhance understanding. A clear reference to this figure within the case study should also be considered.

Done.

2. Results - Figure 2: NADPH is mentioned only in this figure and lacks corresponding references in the main text.

Done - we now explain the contents of figure 2 in the main text.

3. Results - Figure 3 - Detailed Process Flow: Depicting the process flow for specific projects like protein-protein interaction or protein-ligand interaction in Figure 3, and outlining differences when executing various projects would be beneficial. Also, clarifying the values in the figure, such as the color bar ranges, and their meanings would enhance understanding. A dedicated method section can be used to illustrate the processing pipeline.

We elucidated the workflows from the case studies, as we agree they are demonstrative of the powerful analyses facilitated by having these tools connected.

4. Results - Placement of Case Studies: It is recommended to relocate the case studies to the results section for a more logical flow of content.

Done.

5. Discussion - Figure 4: It's unclear how the visualization was achieved. Is this the view from within Foldy?

The visualization was made in PyMol, which is now stated explicitly. We find that complex renders are best done outside of Foldy.

6. Discussion - Figure 5: Details on how membrane proteins were modelled and protein-protein interactions predicted when membrane protein is involved should be provided, especially as PP_0765 and PP_2019 are shown in Figure 5.

Done. This section has been substantially reworded for clarity.

Reviewer #3: This manuscript described a web server that assemble multiple state-of-the-art AI-based tools in structural biology, including AlphaFold and DiffDock. There are also existing web servers providing such services for proteins. For example, Neurosnap (<https://neurosnap.ai/>) provides web services including AlphaFold, DiffDock, ESMFold, LightDock, and etc,. There are even more web servers for protein-ligand binding. The manuscript did not demonstrate the advantages of their own server comparing to the others thoroughly. Besides, the provided application requires a sophisticated local installation to run the web tools instead of providing a directly usable web server, it increased the barrier to access the services to life science researchers. The platforms might be user-friendly, but the way to set up the platforms is not so friendly.

With regard to comparable web servers: we now compare explicitly to other web servers which run AlphaFold. We found that all comparable web servers are proprietary and closed-source, with the exceptions of ColabFold and AlphaFold-Colab which lack many convenience features of Foldy. We believe that Foldy fills an important need in the open source structural biology community.

With regard to the difficulty of setup: we now have a version which can be installed on a blank Google Cloud VM with less than 10 minutes of work, which we believe can improve the broad adoption and applicability of Foldy.
